# Collateral Vessels on 4D CTA as a Predictor of Hemorrhage Transformation After Endovascular Treatments in Patients With Acute Ischemic Stroke: A Single-Center Study

**DOI:** 10.3389/fneur.2020.00060

**Published:** 2020-02-07

**Authors:** Ruoyao Cao, Gengfan Ye, Rui Wang, Lei Xu, Yun Jiang, Guoxuan Wang, Daming Wang, Juan Chen

**Affiliations:** ^1^Beijing Institute of Geriatrics, Beijing Hospital, National Center of Gerontology; Institute of Geriatric Medicine, Chinese Academy of Medical Sciences, Beijing, China; ^2^Department of Radiology, Beijing Hospital, National Center of Gerontology; Institute of Geriatric Medicine, Chinese Academy of Medical Sciences, Beijing, China; ^3^Department of Neurosurgery, Beijing Hospital, National Center of Gerontology; Institute of Geriatric Medicine, Chinese Academy of Medical Sciences, Beijing, China; ^4^Graduate School of Peking Union Medical College, Beijing, China; ^5^Department of Neurology, Beijing Hospital, National Center of Gerontology; Institute of Geriatric Medicine, Chinese Academy of Medical Sciences, Beijing, China

**Keywords:** collateral circulation, hemorrhagic transformation, stroke, endovascular treatment, 4D CTA

## Abstract

**Objective:** Although the benefits of good collateral circulation on infarct volume and outcomes have been confirmed in previous studies, few studies have investigated the relationship between hemorrhagic transformation (HT) and collateral circulation in acute ischemic stroke (AIS). This study aimed to assess whether collateral circulation is an essential factor of HT after endovascular treatments (EVTs).

**Methods:** In total, 71 consecutive AIS patients who underwent EVTs between July 2015 and February 2019 were retrospectively studied. The correlations among HT, collateral vessels on 4D CT angiography (4D CTA), and other predictive factors for HT [e.g., National Institutes of Health Stroke Scale (NIHSS) score, age, sex, serum glucose, and atrial fibrillation history] were evaluated by logistic regression analysis.

**Results:** The rate of hemorrhagic transformation was 42.3% (30/71) in AIS patients. Multivariate logistic regression showed that a good collateral status (OR 0.76, 95% CI 0.73–0.80) was associated with a lower risk of HT. History of atrial fibrillation (OR 2.35, 95% CI 1.96–2.82), baseline NIHSS scores (OR 2.00, 95% CI 1.72–2.32), and higher serum glucose levels (OR 1.70, 95% CI 1.57–1.85) were all independent risk factors of HT.

**Conclusions:** Patients with poor collateral circulation are at a higher risk of HT after receiving endovascular therapy. Thus, variations in collateral circulation based on 4D CTA may be an important factor for personalized clinical treatments. In addition, high blood glucose, atrial fibrillation and the baseline NIHSS score are all important independent predictors of HT.

## Introduction

With the rapid development of endovascular therapeutic techniques and relevant materials, a series of large randomized trials have confirmed that endovascular treatments (EVTs) can significantly reduce the disability and mortality rates of acute ischemic stroke (AIS) patients with intracranial large vessel occlusion (LVO) and markedly improve their prognoses. Thus, EVTs are effective in treating AIS (1–4). However, people have focused more on the complications of EVTs, including postoperative hemorrhagic transformation (HT), vascular reocclusion, and distal embolization. These complications limit the development and use of EVTs to some extent (5). Intracranial HT is a common and severe complication in AIS patients, with an incidence rate as high as 40% (6, 7). Therefore, it is crucial to explore the causes of HT in AIS patients undergoing EVTs.

Recent studies have shown that preoperative use of recombinant tissue-type plasminogen activator (rtPA), diabetes mellitus and a long interval from symptom onset to treatment could increase the occurrence of HT (8). Previous studies have shown that good collateral circulation can predict smaller infarct volume and better clinical recovery after recanalization (9, 10). However, few studies have focused on the association between collateral circulation and HT after EVTs (11, 12). In this study, we aimed to assess whether collateral circulation is an essential factor of HT after EVTs.

## Methods

### Study Population

Data from AIS patients who presented to the emergency department and received EVTs in our hospital from July 2015 to February 2019 were retrospectively analyzed. The local institutional research ethics board approved the study, and informed consent was waived, as the patients' data were evaluated retrospectively and anonymously.

The inclusion criteria were as follows: (1) age ≥ 18 years old; (2) EVTs < 24 h after symptom onset; and (3) large vessel occlusion of the unilateral anterior circulation as observed by one-stop 4D CT angiography (CTA)-CT perfusion (CTP) examination. The exclusion criteria were as follows: (1) intracranial hemorrhage or subarachnoid hemorrhage as observed by a non-contrast CT (NCCT) scan; (2) previous large cerebral infarction of the ipsilateral cerebral hemisphere (infarct size equal to or larger than two-thirds of the middle cerebral artery territory); (3) severe cardiac insufficiency, hepatic insufficiency, pulmonary insufficiency, renal insufficiency and hematological diseases; and (4) history of iodine allergy.

### Data Collection

#### Imaging Data Collection

First, all patients underwent NCCT to exclude intracranial hemorrhage (scan parameters: 120 kV/200 mAs/detector and 0.5 × 80/volume scan). The ASPECTS scoring system (a 10-point scale grading system) was used to evaluate early ischemic changes in the brain parenchyma (13). Then, one-stop whole-brain dynamic volume 4D CTA-CTP examinations (Aquilion ONE, Canon Medical Systems) were performed using 320 × 0.5 mm detector row CT. Intravenous infusion of 40–50 ml non-ionic iodinated contrast medium (iopamidol, Bracco Sine, Shanghai, China) was injected according to iodine concentration per body weight (0.6 ml/kg) followed by 30 ml saline with a two-channel high-pressure injector. A dynamic volume perfusion scan was performed (scan parameters: 80 kV, 100 mAs, coverage area of 160 mm, and layer thickness of 0.5 mm) 7 s after contrast injection. Patients who met the standards for intravenous thrombolysis within the treatment window received rtPA intravenous thrombolysis before EVTs.

Large vessel occlusion and collateral status were obtained from 4D CTA. Collateral circulation was graded based on a 5-point scale: 0 represented no or few collateral vessels (<50% flow of the normal side) on the lesion side in any phase; 1 represented partial collateral vessels (between 50 and 100% flow of the normal side) until the late venous phase; 2 represented partial collateral vessels (between 50% and 100% flow of the normal side) before the venous phase; 3 represented complete collateral circulation (≥100% flow of the normal side) in the late venous phase (whether partial collateral vessels were found before the venous phase or not); and 4 represented complete collateral circulation (≥100% flow of the normal side) before the venous phase. All patients were divided into two groups according to their collateral circulation scores: poor collateral circulation (0–2 points) and good collateral circulation (3–4 points) (14).

The Colt burden score (CBS) was defined as the extent of thromboembolic vessels of the anterior circulation based on CTA. Occlusions of the supraclinoid internal carotid artery (ICA) and proximal and distal M1 segment were counted as 2 points, respectively, while each segment of the M2 branches, anterior cerebral artery and infraclinoid ICA occlusion were counted as 1 point, respectively. If thromboembolism was observed in any segment, the corresponding point value was subtracted from 10. The remaining points constituted the final score (15).

#### Clinical Data Collection

The following information was collected: ① the National Institutes of Health Stroke Scale (NIHSS) score (scale ranges from 0 to 42) (16); ② general information, such as age and gender; ③ the time from symptom onset to puncture and the time from puncture to recanalization; ④ the risk factors of cerebrovascular disease, including history of hypertension, diabetes mellitus, hyperlipidemia, atrial fibrillation and smoking; ⑤ laboratory tests, such as fibrinogen, INR, APTT, PLT, and BNP; and ⑥ the type of stroke based on the Trial of ORG 10172 in Acute Stroke Treatment (TOAST) classification (17).

#### Evaluation of Clinical Outcomes and Adverse Events

Clinical outcomes and adverse events were evaluated based on the following: ① HT was defined as any HT seen on 24 h imaging after EVTs [including petechial hemorrhagic infarction (HI) only seen on MRI]. NCCT or MRI examination performed 24 h after EVTs were used to reveal HT; ② the modified Rankin scale (mRS) score after 3 months was used to evaluate the patients' functional outcomes. The patients were determined to have a good prognosis if the mRS score was 0–2 and a poor prognosis if the mRS score was 3–6 (10).

All baseline and follow-up imaging assessments were performed separately by the same neuroradiologist (RW, 15 years of experience), who was blinded to the clinical outcomes.

### Related Factors for EVTs

EVTs were performed in patients with large vessel occlusions according to the results of one-stop 4D CTA-CTP. For patients undergoing EVTs, conscious sedation or local anesthesia was used according to the patient's condition. EVTs included intra-arterial thrombolysis, stent retriever (SR), contact aspiration (CA), a combination of stent retriever and aspiration (Solumbra), and percutaneous transluminal angioplasty and/or stenting (PTAS). Recanalization of the occluded artery was evaluated on the final angiography and classified according to the modified Thrombolysis In Cerebral Ischemia (mTICI) grade. Successful recanalization was defined as an mTICI grade ≥ 2b (1).

### Statistical Analysis

Normal quantitative data are expressed as the mean ± standard error of the mean (SEM); non-normal quantitative data were expressed as median (Q1, Q3); and qualitative data were expressed as frequency (percentage). Due to the skewness of some collected data, both parametric and non-parametric statistical tests were used. The chi-square test was performed to test qualitative data for differences between the HT group and the non-HT group. The non-parametric Mann-Whitney U test was performed to test non-normal quantitative data for differences between the HT group and the non-HT group. An unpaired *t*-test was performed to test differences in normal quantitative data between the HT group and the non-HT group. Mixed logistic regression was used to analyze the risk factors of HT. The variables in the regression were selected by the Akaike Information Criterion (AIC). Ten-fold cross-validation was performed to train the regression model, which can effectively reduce the occurrence of over-fitting. Receiver operating characteristic (ROC) curve was used to evaluate the different risk factors for predicting the HT. The results were considered significant for two-tailed *p* < 0.05. Statistical analysis was performed using the Statistical Package for the Social Sciences ver. 25 software (SPSS, Chicago, IL).

## Results

### Population Baseline

Overall, the hemorrhage transformation rate was 42.3% (30/71). Of the 71 patients, 38 (53.5%) had atrial fibrillation; 30 (42.3%) had diabetes mellitus; 55 (77.5%) had hypertension; and 22 (30.1%) had coronary heart disease. According to the TOAST classification, the etiological factor for stroke was cardiogenic embolism in 40 (56.3%) patients and large-artery atherosclerosis in 20 (28.1%) patients. Other determined or undetermined reasons for stroke existed for 3 (4.3%) and 8 (11.4%) patients, respectively. Detailed baseline and clinical characteristics are organized in Table 1.

**Table 1:** Baseline clinical and imaging characteristics.

	**HT (*n* = 30)**	**non-HT (*n* = 41)**	***P***
Clinical data			
Age (years), mean ± SD	77.17 ± 8.07	68.85 ± 12.70	<0.001
Male, *n* (%)	15 (50.00)	22 (53.70)	0.761
NIHSS, median (IQR)	18.50 (14.00, 21.50)	14.00 (8.00, 16.00)	<0.001
SBP (mm Hg)	160.57 ± 24.48	139.80 ± 23.87	0.001
DBP (mm Hg)	84.64 ± 13.86	79.95 ± 15.43	0.192
Risk factors, *n* (%)			
AF history	20 (66.70)	18 (43.90)	0.057
Hypertension	26 (86.70)	29 (70.70)	0.110
Diabetes mellitus	14 (46.70)	16 (39.00)	0.520
Hyperlipidemia	18 (60.00)	15 (36.60)	0.051
Smoking	11 (36.70)	14 (34.10)	0.826
Previous stroke	9 (30.00)	13 (31.70)	0.878
Coronary heart disease	19 (63.30)	9 (22.00)	<0.001
Stroke Cause, *n* (%)			0.241
Cardioembolism	20 (66.70)	20 (48.80)	
Large-artery atherosclerosis	8 (26.70)	12 (29.30)	
Other determined/ undetermined etiology	2 (6.70)	9 (21.95)	
Laboratory parameters			
Serum glucose (mmol/L), median (IQR)	7.75 (6.58, 9.63)	7.00 (5.75, 8.75)	0.144
PT (s), median (IQR)	11.20 (10.80, 11.90)	11.30 (10.50, 12.45)	0.944
APTT (s), median (IQR)	31.65 (29.58, 35.10)	33.80 (30.15, 36.50)	0.481
INR, median (IQR)	0.97 (5.94 1.04)	0.98 (0.92, 1.08)	0.898
RBC (×10^12^ cells/L), median (IQR)	4.39 (3.95, 4.78)	4.46 (4.05, 5.00)	0.247
WBC (×10^9^ cells/L), median (IQR)	7.31 (6.27, 9.48)	8.04 (6.03, 10.49)	0.662
Platelet count (×10^9^ cells/L), mean ± SD	185.69 ± 62.31	225.05 ± 100.98	0.063
BNP, median (IQR)	311.51 (185.82, 395.23)	171.59 (53.68, 492.21)	0.279
ALT (IU/L), median (IQR)	14.00 (10.00, 21.00)	16.00 (12.00, 23.50)	0.250
AST (IU/L), median (IQR)	20.50 (16.00, 24.92)	18.00 (15.00, 22.50)	0.299
Creatinine (μmol/L), median (IQR)	78.50 (63.50, 102.00)	67.00 (60.50, 84.00)	0.099
Imaging examination			
ASPECTS, median (IQR)	6.00 (4.00, 8.00)	8.00 (5.50, 8.00)	0.151
HMCAS, median (IQR)	16(53.30)	19(46.30)	0.561
Clot burden score, median (IQR)	3.00 (1.00, 6.00)	9.00 (4.00, 10.00)	<0.001
Collateral score, *n* (%)			0.010
Good collaterals	24 (80.00)	20 (50.00)	
Poor collaterals	6 (20.00)	20 (50.00)	
Occlusion site			0.001
Distal	2 (6.67)	13 (31.71)	
MCA	7 (23.33)	18 (43.90)	
ICA	17 (56.67)	8 (19.51)	
Tandem occlusion (ICA1 MCA M1/M2)	4 (13.33)	2 (4.88)	

*NIHSS, National Institute of Health Stroke Scale; SBP, Systolic blood pressure; DBP, Diastolic blood pressure; AF, atrial fibrillation; PT, Prothrombin time; APTT, stands for Activated partial thromboplastin time; INR, International normalized ratio; RBC, Red blood cells; WBC, White blood cells; BNP, B-natriuretic peptide; ALT, stands for Alanine transaminase; AST, Aspartate transaminase; ASPECTS, Alberta stroke program early CT score; HMCAS, hyperdense middle cerebral artery sign; MCA, middle cerebral artery; ICA, internal carotid artery; IQR, interquartile range*.

The mean time between symptom onset and puncture was 355.21 min (range 183.00–428.00 min), and the time from puncture to recanalization was 89.52 min (range 49.25–123.25 min). The mean number of passes of the thrombectomy device used on each patient were 1.69 (range 1.00–3.00). Seventeen (23.9%) patients received CA; 21 (29.6%) patients were treated with Solumbra; and 23 (32.4%); and 10 (14.1%) patients underwent SR and PTAS treatment, respectively. Patients with a good prognosis showed the low baseline NIHSS scores, and significant higher 4D CTA collateral circulation scores (median = 3, *p* < 0.001) in comparison with those of poor outcomes. More details are shown in Table 2.

**Table 2:** Data of endovascular treatments.

	**HT (*n* = 30)**	**non-HT (*n* = 41)**	***P***
IV rtPA	20 (66.67)	26 (68.42)	0.878
Onset to puncture (min), median (IQR)	257.50 (187.75, 351.49)	303.00 (180.00, 585.57)	0.154
Puncture to recanalization (min), median (IQR)	67.00 (41.00, 120.49)	70.00 (51.50, 120.50)	0.972
mTICI grade			0.842
<2b	6 (20.00)	9 (21.95)	
2b-3	24 (80.00)	32 (78.05)	
Passes of the thrombectomy device			0.887
≤3	26 (86.67)	36 (87.80)	
>3	4 (13.33)	5 (12.20)	
Mode of endovascular treatment, *n* (%)			0.079
CA	10 (33.33)	7 (17.07)	
Solumbra	11 (36.67)	10 (24.39)	
SR	5 (16.67)	18 (43.90)	
PTAS	4 (13.33)	6 (14.63)	
Follow-up infarct volume (52 patients) (ml), mean ± SD	147.07 ± 125.51	61.57 ± 97.07	0.001
Poor outcome (mRS score, 3–6)	23 (76.70)	17 (41.5)	0.003

*IV rtPA, concomitant treatment with intravenous recombinant tissue-type plasminogen activator, mTICI, modified thrombolysis in cerebral infarction scale; CA, contact aspiration; SR, stent retriever; PTAS, percutaneous transluminal angioplasty and/or stenting; mRS, modified rankin scale*.

### Risk Factors of Hemorrhage

**Figure 1:**
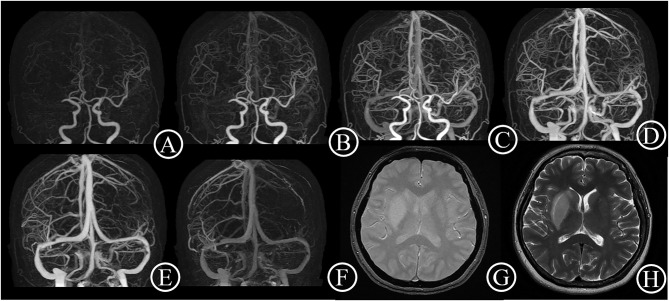
Good collateral case. One 48-year-old female patient suddenly suffered from inability to lift left upper limb for 30 min. She had an initial NIHSS score of 11 and a mRS score of 0 after 3 months. 4D CTA **(A–F)** showed occlusion of right middle cerebral artery, and complete collateral circulation before venous phase. Thus, the patient got a 4D CTA collateral circulation score of 4. The T2 star **(G)** and T2WI **(H)** on day 6 showed a small acute infarct lesion without hemorrhage transformation.

**Figure 2:**
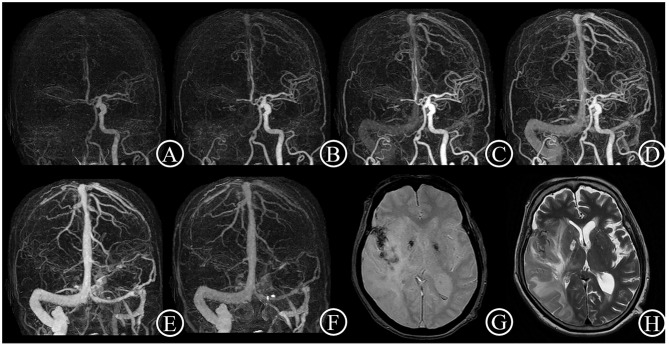
Poor collateral case. One 84-year-old female patient suddenly suffered from inability to lift left upper limb for 1.5 h. She had an initial NIHSS score of 11 and a mRS score of 4 after 3 months. 4D CTA **(A–F)** showed occlusion of right distal carotid artery and right middle cerebral artery, and few collateral vessels in the lesion side in any phase. Thus, the patient got a 4D CTA collateral circulation score of 0. The T2 star **(G)** and T2WI **(H)** on day 7 showed a large acute infarct lesion with hemorrhage transformation.

**Figure 3:**
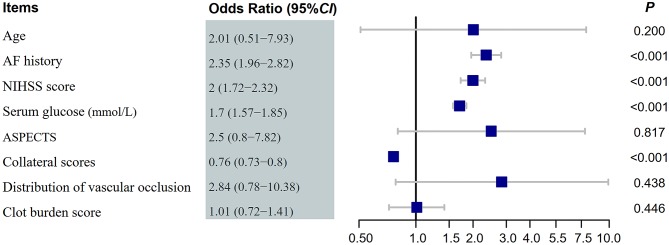
Forest plot of hemorrhage transformation after endovascular treatments. NIHSS, National Institute of Health Stroke Scale; AF, atrial fibrillation; ASPECTS, Alberta stroke program early CT score; OR, odds ratios.

Multivariate logistic regression showed that good collateral status (OR 0.76, 95% CI 0.73–0.80) was associated with a lower risk of HT (Figures 1, 2). Hyperlipidemia (OR 2.35, 95% CI 1.96–2.82), baseline NIHSS scores (OR 2.00, 95% CI 1.72–2.32), and serum glucose (OR 1.70, 95% CI 1.57–1.85) were also independent risk factors of HT. The multivariate logistic regression results are summarized in Table 3 and Figure 3.

**Table 3:** Multivariate logistic regression analysis of risk factors for HT.

	**β coefficient**	**SE**	**Wald χ^2^**	***P***	**OR**	**95%CI**
Age (years)	0.70	0.70	1.64	0.200	2.01	0.51–7.93
AF history	0.85	0.09	32.94	<0.001	2.35	1.96–2.82
NIHSS	0.69	0.08	45.92	<0.001	2.00	1.72–2.32
Serum glucose (mmol/L)	0.53	0.04	76.20	<0.001	1.70	1.57–1.85
ASPECTS	0.92	0.58	0.05	0.817	2.50	0.80–7.82
Collateral score	−0.27	0.02	121.44	<0.001	0.76	0.73–0.80
Occlusion site	1.04	0.66	0.60	0.438	2.84	0.78–10.38
Clot burden score	0.01	0.17	0.581	0.446	1.01	0.72–1.41

*SE, Standard error; OR, odds ratios; CI, confidence interval; AF, atrial fibrillation*.

**Figure 4:**
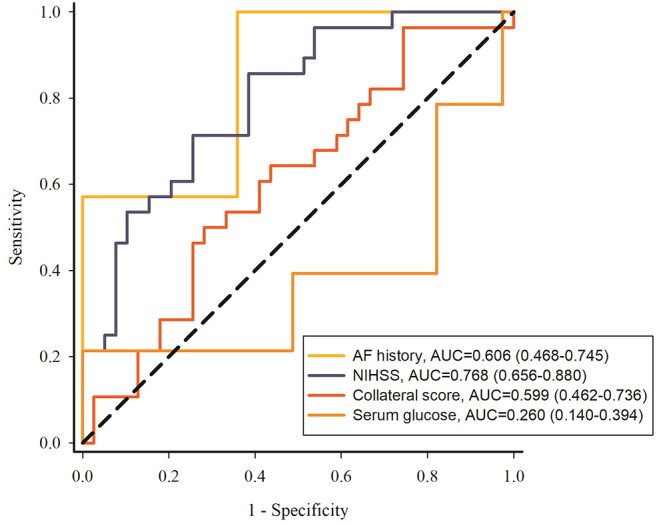
ROC curves of atrial fibrillation history, NIHSS, collateral score and serum glucose to predict the HT. NIHSS, National Institute of Health Stroke Scale; AF, atrial fibrillation.

ROC curves were utilized to evaluate the different risk factors for predicting the HT. The results were shown in Figure 4: NIHSS score was better for predicting the HT (AUC, 0.768; 95% CI: [0.656–0.880]) compared with other factors including atrial fibrillation history (AUC, 0.606; 95% CI: [0.468–0.745]), collateral score (AUC, 0.599; 95% CI: [0.462–0.736]), and serum glucose (AUC, 0.260; 95% CI: [0.140–0.394]).

## Discussion

EVTs have unique advantages for improving the prognosis in AIS patients (1, 18). However, EVTs associated with HT require awareness and careful evaluation. Thus, we performed this single-center retrospective study on AIS patients who underwent EVTs. According to the results of our study, the independent prognostic factors for HT were collateral circulation, baseline NIHSS scores, serum glucose and hyperlipidemia.

### Collateral Circulation

Previous studies have reported that good collateral circulation could reduce infarct volume, attenuate the severity of neurological deficits, and improve the clinical prognosis (9, 10, 19). In this study, we found that the risk of HT was higher in patients with poor collateral circulation than in those with good collateral circulation, even though the occluded vessels were recanalized (Figures 1, 2). The possible reasons are as follows: (1) The blood-brain barrier (BBB) is a natural barrier system for the human brain. It strictly controls the penetration of substances into and out of the brain and thereby maintains the normal physiological activities of the brain tissue and nervous system. The occurrence of HT is closely related to BBB destruction, collateral circulation and ischemia-reperfusion injury. In addition, many studies have indicated that the ischemic penumbra is maintained by collateral flow (20–22). In the case of poor collateral circulation, the penumbra cannot tolerate ischemia, and this intolerance leads to a massive cerebral infarction. The small vessels in the area surrounding the massive cerebral infarction will be compressed when cerebral edema occurs. With the extension of ischemic and hypoxic time, the integrity of the BBB will be damaged. The integrity of the BBB is dependent on the tight junctions of the endothelial cells and the basal lamina. The basal lamina consists of extracellular matrix proteins, including type IV collagen, laminin, coagulation proteins, proteoglycans and fibronectin. The destruction of laminin and fibronectin in the basal lamina may play a role in the pathogenesis of HT (23). In addition, the following reactions occur after reperfusion: endothelial cell adhesion molecule expression, excitatory amino acid release, prostaglandin increase, neutrophil activation and free radical production. These reactions induce endothelial cell damage, increase microvessel permeability, and further exacerbate the destruction of the BBB, which leads to the leakage of red blood cells and other substances from the blood vessels and even HT, although the vessels are recanalized within the treatment window (22, 24). (2) Good collateral circulation could provide forward or reverse compensatory blood flow through the circle of Willis and the leptomeningeal collateral circulation, which help to maintain the blood supply around the infarction core for a relatively long period after the onset of stroke and preserve salvable brain tissue. Two meta-analyses that included more than 20 previous studies systematically reviewed the effects of baseline collateral circulation on clinical and imaging outcomes in AIS patients treated with intra-arterial thrombolysis or mechanical thrombectomy. Both of the meta-analyses indicated that better baseline collateral circulation could improve the proportion of vascular recanalization after EVTs and result in good functional outcomes; in addition, the risk of symptomatic intracranial hemorrhage in the short term (within 7 days or during hospitalization) could be reduced by approximately half (25, 26). As mentioned above, these results may be due to the compensatory effect of the collateral circulation on local blood flow in the event of acute vascular occlusion, which thus help to maintain salvageable brain tissue (ischemic penumbra) around the infarct core. Furthermore, the retrograde collateral circulation may promote clot removal by delivering thrombolytic substances to the clot through retrograde flow, and collateral vessels could create backpressure that helps the mechanical thrombectomy device remove the thrombus. Therefore, good collateral circulation could improve the success rate of reperfusion (27).

To our knowledge, only a few studies have reported that poor collateral circulation is an independent predictor of HT so far, and none of them used 4D CTA to evaluate collateral vessels (11, 12). As a particularly important factor, collateral circulation is directly related to the ischemic penumbra and infarct volume, affects indirectly the integrity of the BBB, and ultimately determines the patient's prognosis and the possibility of HT. Our previous study showed that 4D CTA was an effective and accurate non-invasive method to evaluate the status of the collateral circulation, and the current study showed that patients with good collateral circulation gained more benefits from EVTs than those with poor collateral circulation (14). Based on the results of the current study and our previous works, we found that aggressive EVTs may yield limited benefits and a relatively higher incidence of HT in patients with poor collateral circulation.

### Other Factors

#### High Serum Glucose

A previous study reported that the serum glucose was elevated in 40% of AIS patients. Some patients without a history of diabetes mellitus may suffer from stress hyperglycemia (28). Both stress hyperglycemia and chronic hyperglycemia are significantly associated with symptomatic intracranial hemorrhage (sICH) (29). A hyperglycemic environment can impair cell metabolism and reduce vasoreactivity, which thereby substantially damages the blood-brain barrier and weakens its protective effects, and increase the hemorrhagic risk after vascular recanalization (30). The results of this study also suggest that hyperglycemia was an independent risk factor for HT after vascular recanalization.

#### Baseline NIHSS Score

Kim et al. reported that the incidence of HT decreased with a low baseline NIHSS score. After adjustment of other confounding factors, a higher baseline NIHSS score was an independent risk factor of HT (31). The results of this study were consistent with previous findings. The NIHSS score reflects the degree of neurological deficits. A higher NIHSS score at onset indicates a larger infarct or poorer collateral circulation.

#### Atrial Fibrillation History

Increasingly more studies have shown that atrial fibrillation is a risk factor of HT. Butcher et al. found that atrial fibrillation is an independent predictor of parenchymal hematoma type 2 (PH-2) (32). A meta-analysis on the risk factors of HT after thrombolysis showed that atrial fibrillation was one of the predictors of thrombolysis-related HT (33). A high incidence of HT caused by atrial fibrillation may be attributed to longer thrombosis time in the atria or auricle and a larger thrombus. The abrupt drop of a thrombus may block the proximal artery and result in a relatively larger cerebral infarction. All the above reasons lead to HT when perfusion is restored (34).

The proportion of patients with HT in this study was higher than that in other studies. The main reason may be that the patients were older and had more complications, such as atrial fibrillation and diabetes.

### Limitations

There are some limitations to this study. First, this study was a single-center study with a small sample size. Thus, a subgroup analysis of HT [hemorrhagic infarctions (HIs) and parenchymal hematomas (PHs) according to the European Cooperative Acute Stroke Study (ECASS)] was not performed in this study. Second, using a small sample for model training may result in over-fitting of the model, but we have adopted 10-fold cross-validation to train the regression model, which can effectively reduce the occurrence of over-fitting. In the future, we will expand the sample size to verify the conclusions. Third, this study was a retrospective study. Cases with incomplete data were not included, so there was some selection bias.

## Conclusion

Although EVTs are widely used in the treatment of AIS, the results of this study showed that patients with poor collateral circulation had a higher risk of HT after EVTs. Thus, it is necessary to respect individual variations in collateral circulation and maximize the benefits of EVT. Moreover, high blood glucose, atrial fibrillation and the baseline NIHSS score are also important independent predictors of HT.

## Data Availability Statement

The datasets generated for this study are available on request to the corresponding author.

## Ethics Statement

The studies involving human participants were reviewed and approved by the ethics committee of Beijing Hospital. Written informed consent for participation was not required for this study in accordance with the national legislation and the institutional requirements.

## Author Contributions

RC collected studies and analyzed the data, and drafted and revised the paper. GY collected studies and analyzed the data. RW performed imaging evaluations and monitored the data collection. LX performed clinical evaluations. YJ drafted and revised paper. GW collected and cleaned the data. DW designed this study and revised paper. JC designed the study, monitored the data collection, drafted and revised the paper, and guarantor.

### Conflict of Interest

The authors declare that the research was conducted in the absence of any commercial or financial relationships that could be construed as a potential conflict of interest.
